# Identification and characterization of the chromosomal *yefM-yoeB* toxin-antitoxin system of *Streptococcus suis*

**DOI:** 10.1038/srep13125

**Published:** 2015-08-14

**Authors:** Chengkun Zheng, Jiali Xu, Sujing Ren, Jinquan Li, Miaomiao Xia, Huanchun Chen, Weicheng Bei

**Affiliations:** 1 State Key Laboratory of Agricultural Microbiology, College of Veterinary Medicine, Huazhong Agricultural University, Wuhan, Hubei, 430070, China; 2 Key Laboratory of Development of Veterinary Diagnostic Products, Ministry of Agriculture, Huazhong Agricultural University, Wuhan, Hubei, 430070, China; 3 College of Food Science and Technology, Huazhong Agricultural University, Wuhan, Hubei, 430070, China

## Abstract

Toxin-antitoxin (TA) systems are widely prevalent in the genomes of bacteria and archaea. These modules have been identified in *Escherichia coli* and various other bacteria. However, their presence in the genome of *Streptococcus suis*, an important zoonotic pathogen, has received little attention. In this study, we describe the identification and characterization of a type II TA system, comprising the chromosomal *yefM-yoeB* locus of *S. suis*. The *yefM-yoeB* locus is present in the genome of most serotypes of *S. suis*. Overproduction of *S. suis* YoeB toxin inhibited the growth of *E. coli*, and the toxicity of *S. suis* YoeB could be alleviated by the antitoxin YefM from *S. suis* and *Streptococcus pneumoniae*, but not by *E. coli* YefM. More importantly, introduction of the *S. suis yefM-yoeB* system into *E. coli* could affect cell growth. In a murine infection model, deletion of the *yefM-yoeB* locus had no effect on the virulence of *S. suis* serotype 2. Collectively, our data suggested that the *yefM-yoeB* locus of *S. suis* is an active TA system without the involvement of virulence.

Toxin-antitoxin (TA) systems are widely distributed and highly abundant in the genomes of bacteria and archaea^1^,^2^. Typically, they consist of two genes: one encoding the antitoxin (A) and the other encoding the toxin (T)^3^. The two genes are usually organized as operons^4^. TA systems were initially identified on low copy number plasmids, where they help to maintain plasmid stability by a mechanism called post-segregational killing or genetic addiction^5^,^6^. Currently, TA systems are classified into five types, according to the nature and mode of action of the antitoxin^2^,^7^. In the type I TA system, the antitoxin is an antisense RNA transcribed from the toxin gene, but in the reverse orientation, thus it binds directly to the toxin mRNA and inhibits production of the toxin^1^. The antitoxin of type III TA system is also an RNA, which interacts directly with the toxin and inhibits its toxicity^8^. Type IV and V are two recently reported TA systems, in which the antitoxin either binds to the toxin targets and functions as an antagonist for the toxin toxicity (type IV)^9^ or inhibits the toxin by cleaving its mRNA specifically (type V)^10^. Type II TA systems are pretty prevalent in bacterial chromosomes and have been most extensively studied^1^,^11^. In the type II TA system, the antitoxin and toxin are both proteins. By forming a complex with its cognate toxin, the antitoxin blocks the toxicity of the toxin^1^.

Various roles are proposed for TA systems, including maintenance of plasmid stability, coping with stress, as mediators of programmed cell death and antiphage activity^2^,^12^,^13^,^14^. However, the possibility that TA systems could contribute to bacterial pathogenicity has been largely neglected. *Mycobacterium tuberculosis* possesses a total of 79 TA systems, whereas its nonpathogenic counterpart, *Mycobacterium smegmatis* contains only four^15^. This led to speculation that TA systems may be related to the pathogenicity of pathogens. A recent study reported that TA systems can promote colonization and stress resistance of uropathogenic *E. coli*^16^. In addition, TA modules have been shown to be involved in the virulence of *Salmonella*^17^ and *Leptospira interrogans*^18^. Thus, evidence is increasing that some TA systems are implicated in bacterial pathogenesis and host-pathogen interactions.

*Streptococcus suis* (*S. suis*) is a major swine pathogen causing severe economic losses in the swine industry worldwide^19^. It is also the causative agent in humans in close contact with infected swine or pork by-products^20^. *S. suis* infections in swine lead to a variety of diseases, including meningitis, septicaemia, endocarditis, arthritis and even sudden death. In humans, the infection causes meningitis, septicaemia and streptococcal toxic shock syndrome (STSS)^19^,^21^. To date, 33 serotypes (types 1 to 31, 33, and 1/2) of *S. suis* have been described based on the composition of the capsular polysaccharide (CPS)^22^,^23^. Among them, *S. suis* serotype 2 (*S. suis* 2 or SS2) is the most virulent and the most commonly isolated serotype in association with diseases in most countries^22^. In 1998 and 2005, two large outbreaks of human *S. suis* 2 infections occurred in China, resulting in 25 cases with 14 deaths and 215 cases with 38 deaths, respectively^21^. Despite numerous studies having been carried out over the past few years, knowledge of the pathogenesis of *S. suis* remains very limited. In the genome of *S. suis* 2 strain SC84, nine type II TA systems have been predicted by bioinformatics analysis (unpublished data). Among them, only SezAT has been identified as an active TA system, yet its functions remain elusive^24^. To gain further insight into the pathogenesis of *S. suis* infection, other TA systems should be identified and their roles should be explored.

The *yefM-yoeB* module belongs to one of the well-characterized TA systems in *E. coli* and *S. pneumoniae*^25^,^26^. This TA module was first identified in *E. coli*, as the homolog of the *Enterococcus faecium axe-txe* TA system^27^. Since then, the *yefM-yoeB* TA system has been described in many bacterial species, including major pathogens such as *S. pneumoniae*^28^,^29^, *M. tuberculosis*^30^ and *Staphylococcus aureus*^31^,^32^. Moreover, the *yefM-yoeB* locus was identified as the first TA system in *Streptomyces*^33^. Recently, the expression pattern and structural peculiarities of the *yefM-yoeB* TA system were investigated in *Lactobacillus rhamnosus*^34^. *E. coli* YefM and YoeB form a heterotrimeric complex (YefM_2_-YoeB) that blocks the effect of the toxin^35^. When liberated from the complex, YoeB binds to the 50 S ribosomal subunit and cleaves mRNA at the A site, thereby inhibiting translation initiation in *E. coli* and *S. aureus*^31^,^36^. Overproduction of the Lon protease specifically activates YoeB-dependent mRNAs cleavage, suggesting that Lon might be responsible for YefM degradation^37^. In addition, the *yefM-yoeB* TA system is transcriptionally autoregulated. YefM can bind to the promoter and repress transcription, while YoeB acts as a repression enhancer^38^. Bioinformatics analysis revealed that the *yefM-yoeB* locus is also present in the genome of most serotypes of *S. suis*. However, whether this locus encodes an active TA system is still unknown.

In this study, a selective expression vector was constructed to characterize the TA systems in *E. coli*. We showed that the *yefM-yoeB* locus is an active TA system of *S. suis*, and introduction of this system into *E. coli* could affect cell growth. We also explored the involvement of *yefM-yoeB* in the virulence of *S. suis* 2 and found that the module has no role in virulence.

## Results

### Identification of the *yefM-yoeB* locus in *S. suis*

**Figure 1:**
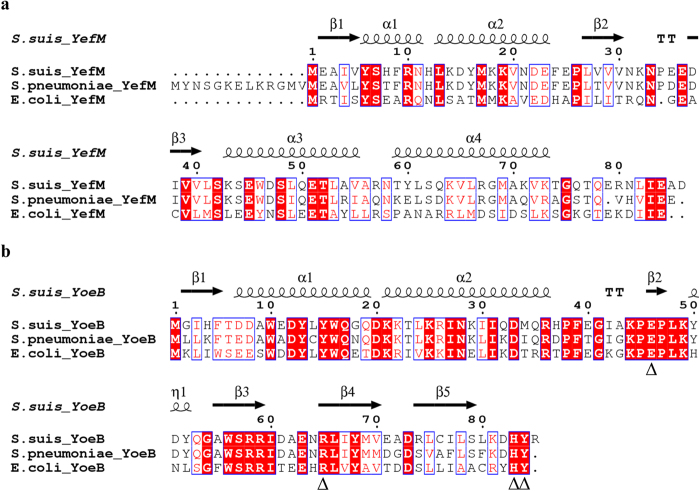
Multiple sequence alignments of the *S. suis* YefM-YoeB system with related homologs. The image was generated using program ESPript 3.0 (http://espript.ibcp.fr/ESPript/cgi-bin/ESPript.cgi). Identical residues are shown as white letters with red background, and similar residues are shown as red letters with white background. The predicted secondary structures of *S. suis* YefM and YoeB are shown at the top. α: α-helix; β: β-sheet; η: coil; T: turn. (**a**) Alignment of the YefM protein family. The GenBank accession numbers are as follows: *S. suis* YefM, YP_003025797.1; *S. pneumoniae* YefM, NP_359178.1; and *E. coli* YefM, NP_416521.2. (**b**) Alignment of the YoeB protein family. The conserved residues required for YoeB activity are labelled with triangles (Δ). The GenBank accession numbers are as follows: *S. suis* YoeB, YP_003025796.1; *S. pneumoniae* YoeB, NP_359177.1; and *E. coli* YoeB, YP_588458.1.

To identify the YoeB toxin homologs, a BlastP search against the proteins annotated in the genome of *S. suis* SC84^39^ was performed, using the YoeB sequences of *S. pneumoniae* R6 and *E. coli* MG1655 as query sequences. Both searches revealed an open reading frame (SSUSC84_1817) encoding an 85 amino acid protein sharing 75% and 50% identity with the *S. pneumoniae* and *E. coli* YoeB toxins, respectively. Hence, the protein was termed YoeB. The YefM antitoxin encoded by SSUSC84_1818 was identified by the same method. BlastP analysis showed that the *S. suis* YefM has 79% and 29% amino acid sequence identity with YefM from *S. pneumoniae* and *E. coli*, respectively. Multiple sequence alignments further revealed that 1) the *S. suis* YefM-YoeB system shares high level of homology with that from *S. pneumoniae* and *E. coli*; 2) YoeB processes several conserved residues required for its activity (Glu46, Arg65, His83 and Tyr84)^35^ (Fig. 1). Protein homology modeling using CPHmodels predicted the structures of *S. suis* YefM and YoeB. The secondary structure of YefM is proposed to consist of four α-helices and three β-sheets (Fig. 1a), while that of YoeB contains two α-helices, five β-sheets and a coil (Fig. 1b). To determine whether the *yefM-yoeB* locus was universally present in the genomes of *S. suis*, BlastN analysis was performed using the 21 complete genomes of *S. suis* available in the National Centre for Biotechnology Information database as of 31 May 2015. The results confirmed that all strains harbour the *yefM-yoeB* locus, except for strain D12, a serotype 9 *S. suis* (see Supplementary Fig. S1 online).

**Figure 2:**
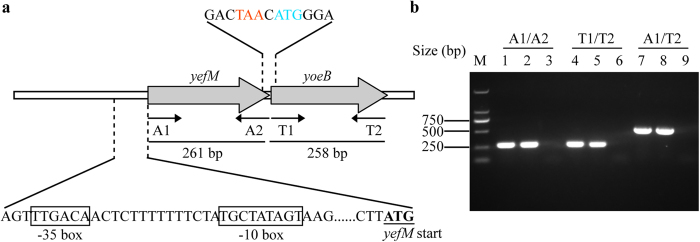
The yefM-yoeB locus is organized as an operon. (**a**) Genetic organization of the *yefM-yoeB* locus in *S. suis* strain SC84. The putative −35 and −10 regions are boxed. The primers used for RT-PCR or PCR are drawn as arrows, and the expected sizes of the corresponding PCR products are shown below. (**b**) Co-transcription analysis. Total RNAs extracted from *S. suis* SC84 were used to synthesize cDNAs. PCR was carried out with primer pairs A1/A2, T1/T2 and A1/T2, respectively. Lanes 1, 4 and 7 represent the amplification using cDNAs as the template; Lanes 2, 5 and 8 represent the amplification using genomic DNA (gDNA) as the template; Lanes 3, 6 and 9 represent the amplification using cDNA- (cDNA reaction without reverse transcriptase) as the template. The DL 2000 DNA Marker is shown on the left (lane M).

A genetic structure analysis revealed that *yefM* is located upstream of *yoeB*, and the two genes are separated by one nucleotide, apparently arranged in a bicistronic operon (Fig. 2a). BPROM analysis of the upstream region of the *yefM* gene (about 300 bp) identified the putative −35 and −10 regions, which are located in the intergenic region between the *yefM* gene and its upstream gene (Fig. 2a). To assess whether *yefM* and *yoeB* are co-transcribed in *S. suis*, a reverse transcription polymerase chain reaction (RT-PCR) analysis was performed. Reverse transcriptase was used to synthesize cDNAs and the resulting cDNAs were PCR amplified using primer pair A1/T2. The individual *yefM* and *yoeB* genes were also amplified using primer pairs A1/A2 and T1/T2, respectively. As shown in Fig. 2b, the PCR products were of the expected sizes for *yefM* (261 bp), *yoeB* (258 bp) and *yefM-yoeB* (520 bp), all consistent with that of the genomic DNA. No PCR products were evident in the negative controls, in which the reverse transcription was performed without the enzyme, therefore eliminating possible DNA contamination. These results demonstrated that in *S. suis*, *yefM* and *yoeB* are actively co-transcribed, thus forming a bicistronic operon.

### Construction of a selective expression vector to characterize the toxin-antitoxin systems in *E. coli*

To characterize the toxin-antitoxin systems in *E. coli*, a selective expression vector was constructed as previously described^40^. A DNA fragment containing the *araC* gene and the promoter *P*_*BAD*_ was amplified from the pBADhisA plasmid, digested with the *Xho* I and *Hin*d III enzymes, and then cloned into pET-30a, an expression vector induced by isopropyl β-D-thiogalactopyranoside (IPTG), to generate the selective expression vector, designated pETBAD (see Supplementary Fig. S2 online). Plasmid pETBAD has five unique restriction sites for cloning and possesses the IPTG-inducible promoter *P*_*lac*_ and the arabinose-inducible promoter *P*_*BAD*_, thus expression can be induced by IPTG and/or arabinose (see Supplementary Fig. S2 online).

### Overproduction of YoeB inhibits cell growth in *E. coli* which can be alleviated by YefM

**Figure 3:**
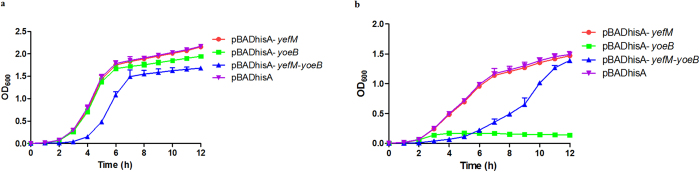
Effect of induction of YoeB, YefM or YefM-YoeB on growth of *E. coli*. Overnight cultures of *E. coli* Top10 cells harbouring the plasmid pBADhisA-*yefM*, pBADhisA-*yoeB*, pBADhisA-*yefM-yoeB* and pBADhisA were diluted 1:1000 in LB-ampicillin. Each culture was then divided into two equal volumes. The first half served as the control, to which 0.2% D-glucose was added (**a**), 0.2% L-arabinose was added to the second half to induce expression of the target gene (**b**). Culture growth was evaluated by measuring the OD_600_ every hour. The data shown are averages with standard deviations for the results from three independent experiments.

To determine whether the *yefM-yoeB* locus is indeed an active TA system, the *yefM* and *yoeB* genes were cloned separately as well as together into the pBADhisA expression vector. The plasmids were introduced into *E. coli* Top10 cells, and the transformants were selected in LB agar plates with 0.2% D-glucose (repressed conditions of *P*_*BAD*_). *E. coli* Top10 cells harbouring the corresponding plasmids were grown in LB medium, and 0.2% D-glucose or L-arabinose was added at time zero. In the presence of 0.2% D-glucose, Top10 cells harbouring the pBADhisA-*yefM* and pBADhisA plasmids showed no major difference in growth, while cells carrying the pBADhisA-yoeB plasmid showed a moderate growth defect (Fig. 3a). In the case of inductive conditions (0.2% L-arabinose), *E. coli* Top10 cells harbouring the pBADhisA-*yoeB* plasmid exhibited drastic growth inhibition, while cells harbouring other two plasmids showed only moderate reductions in their OD_600_ value (Fig. 3b). Surprisingly, under both repressed and inductive conditions, *E. coli* Top10 cells harbouring the pBADhisA-*yefM-yoeB* plasmid exhibited obvious growth inhibition (Fig. 3), Even so, Top10 cells carrying the pBADhisA-*yefM-yoeB* plasmid showed much better growth than that carrying pBADhisA-*yoeB* (Fig. 3b), indicating that YoeB-induced growth inhibition could be alleviated by YefM.

**Figure 4:**
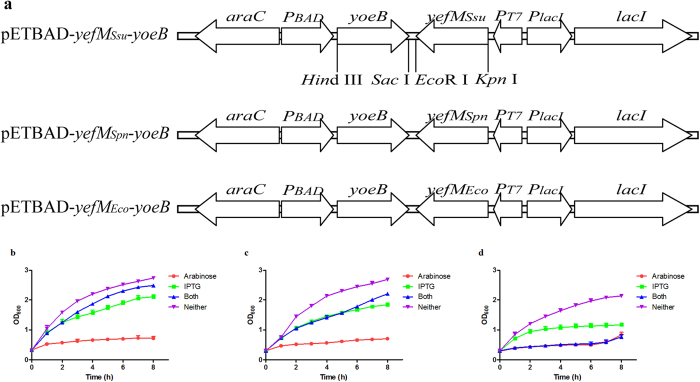
Toxicity of YoeB_*Ssu*_ can be alleviated by YefM_*Ssu*_ and YefM_*Spn*_, but not by YefM_*Eco*_. *E*. *coli* BL21(DE3) cells harbouring the respective selective expression plasmids were grown to an OD_600_ of about 0.3. Each culture was then divided into four equal parts, to three of which were individually added 0.2% L-arabinose, 1 mM IPTG or both, respectively. The fourth part served as the control, to which nothing was added. These cultures were further incubated and samples were taken every hour to determine the OD_600_. (**a**) Schematic representation of plasmids for the selective expression of YefM and YoeB under the control of *P*_*BAD*_ and *P*_*lac*_, respectively. (**b**) Toxicity of YoeB_*Ssu*_ can be alleviated by YefM_*Ssu*_. (**c**) Toxi**c**ity of YoeB_*Ssu*_ can be alleviated by YefM_*Spn*_. (**d**) Toxicity of YoeB_*Ssu*_ cannot be alleviated by YefM_*Eco*_. The data shown are averages with standard deviations for the results from three independent experiments.

We further investigated the toxic and antitoxic effect of the TA components using the selective expression system constructed here. In the selective expression plasmid, the IPTG-inducible promoter *P*_*lac*_ and the arabinose-inducible promoter *P*_*BAD*_ control the expression of YefM and YoeB, respectively (Fig. 4a). Thus, *E. coli* BL21 (DE3) cells harbouring the pETBAD-*yefM*_*Ssu*_*-yoeB* plasmid could express the *S. suis* YefM and/or YoeB upon induction with IPTG and/or L-arabinose. As shown in Fig. 4b, the *E. coli* BL21 (DE3) cells exhibited considerable growth inhibition in the presence of L-arabinose. In contrast, only moderate growth inhibition was observed in the presence of IPTG or IPTG and L-arabinose together.

These results indicated that the protein encoded by the *yoeB* gene is a toxin against *E. coli* and that the protein encoded by the *yefM* gene could counteract the toxicity. Therefore, the *S. suis yefM-yoeB* locus works as a typical TA system.

### YoeB_*Ssu*_-induced growth inhibition in *E. coli* that could be alleviated by YefM_*Spn*_, but not by YefM_*Eco*_

In *S. pneumoniae* and *E. coli*, the toxicity of YoeB could be counteracted only by its cognate antitoxin^28^. To test whether there was cross-complementation between non-cognate YefM and the *S. suis* YoeB, *E. coli* BL21 (DE3) cells were transformed with plasmid pETBAD-*yefM*_*Spn*_*-yoeB* and pETBAD-*yefM*_*Eco*_*-yoeB* (Fig. 4a). As seen in Fig. 4c, induction of YoeB_*Ssu*_ resulted in a drastic reduction in OD_600_ value, whereas coinduction of YefM_*Spn*_ and YoeB_*Ssu*_ alleviated the growth inhibition in *E. coli*, indicating that the toxic effect of YoeB_*Ssu*_ was counteracted by coexpression of YefM_*Spn*_. However, coinduction of YefM_*Eco*_ and YoeB_*Ssu*_ did not neutralize the YoeB_Ssu_ toxicity (Fig. 4d). In addition, induction of YefM_*Ssu*_, YefM_*Spn*_ and YefM_*Eco*_ also displayed an effect on growth inhibition in *E. coli* (Fig. 4b–d).

### Introduction of the *S. suis yefM-yoeB* system into *E. coli* could affect cell growth

**Figure 5:**
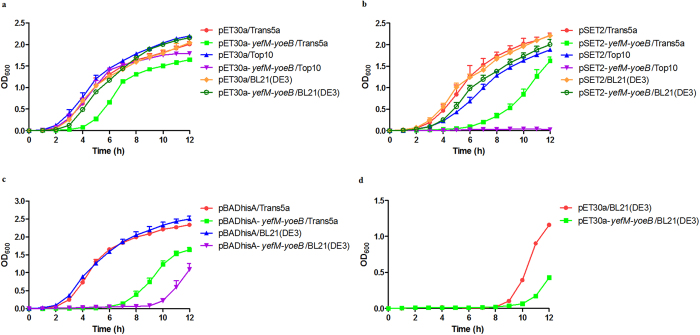
Effect of introduction of the *S. suis yefM-yoeB* system into *E. coli*. (**a**) Growth curves of *E. coli* strains carrying the pET30a-*yefM-yoeB* or pET-30a plasmid. Cells were grown in LB-kanamycin. (**b**) Growth curves of *E. coli* strains carrying the pSET2-*yefM-yoeB* or pSET2 plasmid. Cells were grown in LB-spectinomycin. (**c**) Growth curves of *E. coli* strains carrying the pBADhisA-*yefM-yoeB* or pBADhisA plasmid. Cells were grown in LB medium-ampicillin. (**d**) Growth curves of *E. coli* BL21(DE3) strain carrying the pET30a-*yefM-yoeB* or pET-30a plasmid. Cells were grown in LB-kanamycin and 1 mM IPTG. The data shown are averages with standard deviations for the results from three independent experiments.

It seemed likely that introduction of the *S. suis yefM-yoeB* system into *E. coli* affects cell growth, since *E. coli* Top 10 cells carrying the pBADhisA-*yefM-yoeB* plasmid showed considerable growth inhibition under both repressed and inductive conditions. To test the hypothesis, the *yefM* and *yoeB* genes were cloned together into the pET-30a expression plasmid. When introduction of the pET30a-*yefM-yoeB* and pET-30a plasmids into *E. coli* Trans5α and Top10 strains, cells carrying pET30a-*yefM-yoeB* showed an obvious growth defect compared with cells carrying pET-30a (Fig. 5a). However, when introduction of the two plasmids into *E. coli* BL21(DE3) strain, no major difference in growth was found (Fig. 5a).

The same experiments were carried out with the pSET2-*yefM-yoeB* plasmid. In this plasmid, the *S. suis yefM-yoeB* locus is under the control of its own promoter. As shown in Fig. 5b, *E. coli* Trans5α cells harbouring the pSET2-*yefM-yoeB* plasmid exhibited a remarkable growth defect compared with that harbouring the empty plasmid. The growth inhibition effect was even more severe when the plasmid was transformed into Top10 strain, as cells carrying the pSET2-*yefM-yoeB* plasmid showed growth arrest over a period of 12 hours (Fig. 5b). However, BL21(DE3) strain harbouring pSET2-*yefM-yoeB* showed only a slight defect in growth.

Next, the pBADhisA-*yefM-yoeB* and pBADhisA plasmids were transformed into *E. coli* Trans5α and BL21(DE3) strains. Both strains carrying the pBADhisA-*yefM-yoeB* plasmid exhibited an obvious defect in growth compared with that carrying the pBADhisA plasmid (Fig. 5c).

Finally, growth of *E. coli* BL21(DE3) strain harbouring the pET30a-*yefM-yoeB* and pET-30a plasmids was determined in the presence of 1 mM IPTG. Under inductive conditions, BL21(DE3) cells harbouring pET30a-*yefM-yoeB* also displayed an obvious growth inhibition effect (Fig. 5d).

Taken together, the results clearly demonstrated that introduction of the *S. suis yefM-yoeB* system into *E. coli* could affect cell growth.

### Construction and microbiological characterization of the Δ*yefM-yoeB* mutant

**Figure 6:**
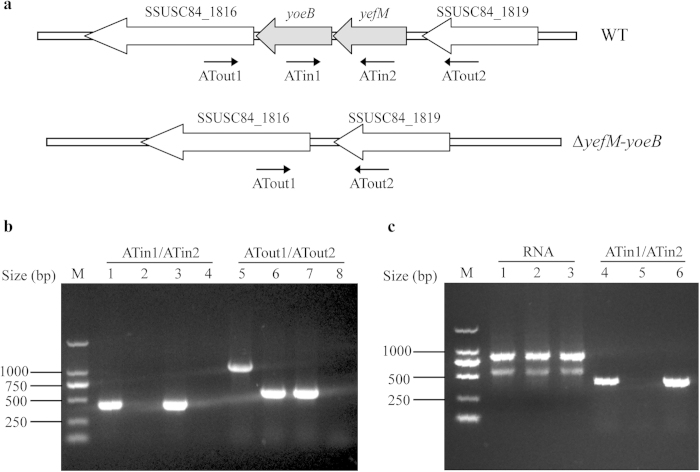
Construction and confirmation of the mutant and complementation strains. (**a**) Di**a**gram of genetic organization of the WT and Δ*yefM-yoeB* strains. (**b**) PCR confirmation of the mutant and complementation strains. The primer pairs used in the PCR analysis are indicated above the lanes. Templates were genomic DNAs from the WT strain (lanes 1 and 5), Δ*yefM-yoeB* (lanes 2 and 6), CΔ*yefM-yoeB* (lanes 3 and 7), and H_2_O (lanes 4 and 8). (**c**) RT-PCR analysis of *yefM-yoeB* transcripts. Total RNAs were extracted from the WT strain (lane 1), Δ*yefM-yoeB* (lanes 2), and CΔ*yefM-yoeB* (lane 3). cDNA generated from these RNA samples was subjected to RT-PCR analysis with primer pair ATin1/ATin2. The products were analysed by electrophoresis (lanes 4, the WT strain; lanes 5, Δ*yefM-yoeB*; lanes 6, CΔ*yefM-yoeB*). The DL 2000 DNA Marker is shown on the left (lane M).

To investigate the functions of the *yefM-yoeB* locus in *S. suis* 2, an isogenic *yefM-yoeB* knockout mutant of *S. suis* 2 strain SC19, termed Δ*yefM-yoeB*, was constructed through homologous recombination (Fig. 6a). To rule out the possible polar effect and introduction of a second mutation during the construction of Δ*yefM-yoeB*, we generated a complementation strain, designated CΔ*yefM-yoeB* using the *E. coli-S. suis* shuttle vector pSET2^41^. The resulting mutant and complementation strains were confirmed by PCR (Fig. 6b), RT-PCR (Fig. 6c) and direct DNA sequencing (data not shown).

**Figure 7:**
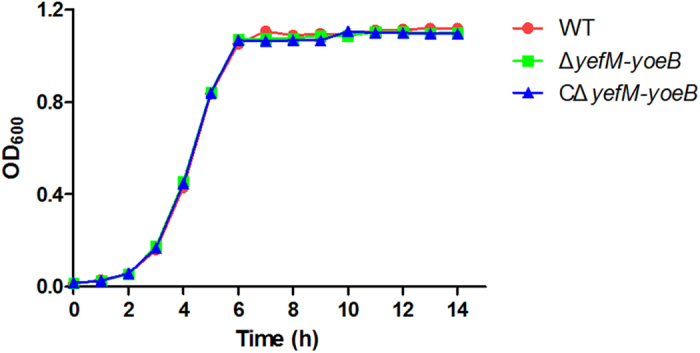
Growth curves of the WT, Δ*yefM-yoeB* and CΔ*yefM-yoeB* strains. The WT, Δ*yefM-yoeB* and CΔ*yefM-yoeB* strains were grown in TSB with 10% newborn bovine serum at 37 °C under static conditions. Samples were taken every hour to measure the OD_600_. The data shown are averages for the results from three independent experiments.

The effects of *yefM-yoeB* deletion on the basic microbiological properties of *S. suis* 2 were investigated in terms of morphology, haemolytic activity and *in vitro* growth. The cell morphologies of the Δ*yefM-yoeB* mutant, WT and CΔ*yefM-yoeB* strains were examined under light microscope using Gram staining. However, no obvious differences were found (see Supplementary Fig. S3a online). When inoculated on sheep blood agar plates, the three strains showed similar haemolytic activity (see Supplementary Fig. S3b online). The growth kinetics of the mutant strain were compared with those of the WT and complementation strains by measuring the optical density at 600 nm (OD_600_) every hour. We found that the growth kinetics of Δ*yefM-yoeB* were essentially identical to those of the WT and CΔ*yefM-yoeB* strains (Fig. 7), indicating that the *yefM-yoeB* locus of *S. suis* 2 plays no role in growth *in vitro*.

### Deletion of the *yefM-yoeB* locus has no effect on *S. suis* 2 virulence in mice

**Figure 8:**
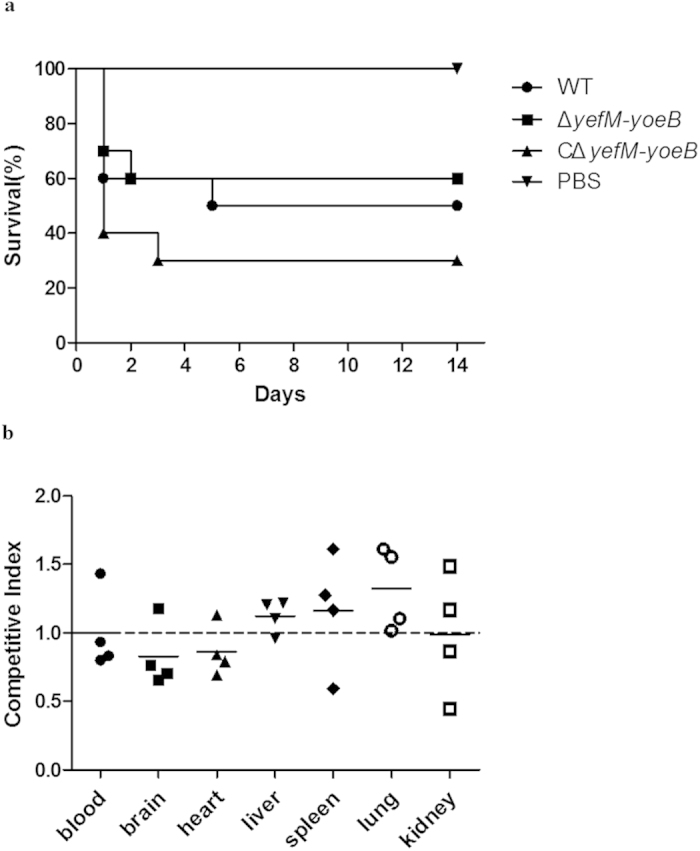
No apparent role of the *yefM-yoeB* locus in the virulence of *S. suis* 2. (**a**) Survival curves of mice infected with the WT, Δ*yefM-yoeB* and CΔ*yefM-yoeB* strains. Groups of ten female CD1 mice were inoculated intraperitoneally with the WT, Δ*yefM-yoeB* and CΔ*yefM-yoeB* strains at a dose of 6 × 10^8^ CFU. Mice inoculated with PBS served as the control. Survival data were analysed with the log-rank test. No significant difference was observed between the Δ*yefM-yoeB* group and the WT group or the CΔ*yefM-yoeB* group. (**b**) Competitive index of Δ*yefM-yoeB* against the WT strain. Four female CD1 mice were inoculated intraperitoneally with a mixture of Δ*yefM-yoeB* and WT at a ratio of 1:1. At 24 h post-infection, blood, brain, heart, liver, spleen, lung and kidney samples from the mice were collected and plated. The Δ*yefM-yoeB*:WT ratio in these samples was determined by analysing 70–90 colonies by colony PCR. The competitive index (CI) was determined as the mutant:WT ratio in the samples divided by the ratio in the inoculum. A CI value of 1 indicates equal competitiveness. Mean CI values from four mice were compared to 1 using the two-tailed paired *t* tes*t* to determine whether the difference in competitiveness was significant. No statistically significant difference was found.

To assess the role of the *yefM-yoeB* locus in the pathogenesis of *S. suis* 2, we performed an experimental infection model in CD1 mice. As an initial comparison of virulence, groups of ten CD1 mice were inoculated intraperitoneally with 6 × 10^8^ CFU of the WT, Δ*yefM-yoeB*, CΔ*yefM-yoeB* strains or PBS. Most mice infected with *S. suis* strains developed typical clinical signs of *S. suis* 2 infection, including depression, lethargy, weakness, prostration and rough coat hair during the first 72 h post infection. Ultimately, the survival rates of mice in the WT, Δ*yefM-yoeB* and CΔ*yefM-yoeB* groups were 50%, 60% and 30%, respectively (Fig. 8a). By contrast, all mice inoculated with PBS remained healthy and survived. No significant difference in survival rates was observed between the Δ*yefM-yoeB* group and the WT group (P = 0.6793), or the C*∆yefM-yoeB* group (P = 0.1924). Thus, it seemed likely that the *yefM-yoeB* locus has no role in *S. suis* 2 virulence.

A competitive-infection assay was adopted to further compare the abilities of the WT strain and the Δ*yefM-yoeB* mutant to establish infection. Four mice were inoculated intraperitoneally with a 1:1 mixture of the WT and mutant bacteria. Mice were sacrificed to collect blood, brain, heart, liver, spleen, lung and kidney samples 24 h after inoculation. Bacterial cells recovered from various tissue samples were analysed by colony PCR to determine the competitive index (CI). The result showed that for each tissue, the mean CI values were approximately 1 (Fig. 8b), suggesting that the mutant and WT strains have similar abilities to colonize the tissues.

Taken together, these results indicated that the *yefM-yoeB* locus is not involved in the virulence of *S. suis* 2.

## Discussion

TA systems have attracted an increasing concern in recent years because of their abundance in the genomes of bacteria and archaea on the one hand, and the limited of knowledge of their physiological functions on the other. In *E. coli* K12, at least 33 TA systems have been identified, with several being well characterized^1^. However, only one TA module, SezAT, has been described in *S. suis*, yet its function has not been demonstrated^24^. The *yefM-yoeB* module is one of the best studied TA systems and has been described in various bacteria, including *E. coli*^27^,^37^, *S. pneumoniae*^28^,^29^, *M. tuberculosis*^30^, *S. aureus*^31^,^32^, *Staphylococcus equorum*^42^ and *Streptomyces*^33^.

In this study, we showed that the chromosomally encoded *yefM-yoeB* locus of *S. suis* is an active TA system with *yoeB* encoding the toxin and *yefM* encoding the cognate antitoxin. This is not surprising, as this system shows considerable similarity to the YefM-YoeB system from *S. pneumoniae* and *E. coli*. Like most TA systems, the *yefM* and *yoeB* genes are co-transcribed. Upstream of the *yefM* gene, there is an intergenic region of 75 nucleotides, which may act as the promoter region. Overproduction of the YoeB toxin in *E. coli* Top10 and BL21 (DE3) cells both resulted in toxic effects commonly linked to toxin activity. YoeB homologs were identified as endoribonucleases that inhibit translation by cleaving mRNA, either in a ribosome-dependent or -independent manner^32^,^35^,^43^. We therefore reasoned that *S. suis* YoeB inhibits cell growth via a similar mechanism.

The toxic effect of *S. suis* YoeB toxin could be neutralized by both the cognate YefM_*Ssu*_ and the heterologous YefM_*Spn*_, but not by the *E. coli* counterpart, consistent with the fact that YefM_*Ssu*_ shares higher levels of identity with YefM_*Spn*_ (79% identity versus 29% for YefM_*Eco*_). As YefM_*Spn*_ and YefM_*Ssu*_ display a high level of sequence homology, we speculated that YefM_*Spn*_ could bind to and neutralize the YoeB_*Ssu*_. The lack of cross-complementation between YefM_*Eco*_ and YoeB_*Ssu*_ suggested that there is no favourable interaction between the two heterologous proteins. Similar behaviour has also been reported for YoeB_*Eco*_, whose activity could be alleviated by both the cognate YefM_*Eco*_ and the antitoxin Axe of *E. faecium*, but not by YefM_*Spn*_^27^,^28^. A previous study showed that overexpression of YefM_*Eco*_ displayed toxicity in *E. coli* at high expression levels^44^, consistent with our observation that YefM_*Ssu*_, YefM_*Spn*_ and YefM_*Eco*_ had an effect on growth inhibition in *E. coli*.

An interesting observation was that *E. coli* Top10 cells harbouring the pBADhisA-*yefM-yoeB* plasmid showed an obvious growth defect under both repressed and inductive conditions. Similar experiments were then performed with other plasmids and strains. Plasmids containing the *S. suis yefM-yoeB* system were introduced into different strains of *E. coli*. Except for BL21(DE3) strain carrying the pET30a-*yefM-yoeB* plasmid, all tested strains harbouring the plasmids containing the *S. suis yefM-yoeB* system showed growth inhibition. A previous study revealed that the YefM-YoeB complex forms a 2:1 heterotrimer^35^. We speculated that the *yefM-yoeB* system could be expressed even under repressed conditions and that YefM expression was not enough to counteract YoeB, thus leading to growth inhibition in *E. coli*. Since pET-30a and *E. coli* BL21(DE3) strain constitute a precise inducible expression system, it is possible that the *yefM-yoeB* locus on pET-30a was not expressed without inducer, therefore growth inhibition was not observed for BL21(DE3) carrying pET30a-*yefM-yoeB* under normal growth conditions. The speculation agrees with the result that BL21(DE3) carrying the pET30a-*yefM-yoeB* plasmid exhibited a growth defect under inductive conditions.

To investigate the functions of the *yefM-yoeB* locus in *S. suis* 2, a knockout mutant and the corresponding complementation strain were constructed. No obvious differences between the WT and the *yefM-yoeB* deletion mutant were found in terms of their cell morphology, haemolytic activity on blood agar plates, and *in vitro* growth. The potential role of TA systems in bacterial pathogenesis has been neglected for a long time. More and more studies have revealed that TA systems are involved in bacterial pathogenicity and host-pathogen interactions^17^,^18^; therefore, we evaluated the effect of this TA system on the pathogenesis of *S. suis* 2 using a murine infection model. Survival curves of mice and the competitive-infection assay both demonstrated that deletion of the *yefM-yoeB* locus had no role in the pathogenicity of *S. suis* 2. It was reported that *Yersinia pestis* lacking the *hicB3* antitoxin is virulence-attenuated; however, the mutant lacking the whole *hicA3B3* locus is fully virulent^45^. In contrast, the toxins ChpK and MazF but not the antitoxins ChpI and MazE are involved in the virulence of *L. interrogans* during infection^18^. Future experiments should evaluate the involvement of the individual genes of the *yefM-yoeB* locus in the virulence of *S. suis* 2.

It should be noted that TA systems play important roles in the physiology of cells, including biofilm formation and multidrug resistance^25^. The effect of the *yefM-yoeB* module on biofilm formation, stress tolerance and formation of persister cells will be explored in future studies. It is proposed that TA systems are potential targets for antibiotics^46^. Given the fact that *S. suis* YoeB can inhibit the growth of *E. coli* considerably, we are planning to examine the effect of YoeB on *S. suis*. If a similar toxic effect is observed, a multivalent strategy to synthesize an inhibitor that interacts with the YefM antitoxin and frees the toxin YoeB to inhibit bacterial growth could be promising for the development of new antibiotics.

In conclusion, the *yefM-yoeB* locus was identified as a new TA system of *S. suis*. The present study clearly demonstrated that the *yoeB* gene encodes a toxin that can inhibit the growth of *E. coli*. Specifically, the toxicity of *S. suis* YoeB could be alleviated by the cognate *S. suis* YefM and heterologous *S. pneumoniae* YefM. More importantly, we reported that introduction of the *yefM-yoeB* TA system into *E. coli* could affect cell growth. In addition, deletion of the *yefM-yoeB* locus had no effect on the virulence of *S. suis* 2.

## Methods

### Bacterial strains and growth conditions

Bacterial strains and plasmids used in this study are listed in Supplementary Table S1. *S. suis* strains were maintained on Tryptic Soy Broth (TSB) or Tryptic Soy Agar (TSA; Difco Laboratories, Detroit, MI, USA) with 10% (vol/vol) newborn bovine serum at 37 °C, unless otherwise specified. *E. coli* strains were cultured in Luria-Bertani (LB) broth or on LB agar at 37 °C. When necessary, antibiotics (purchased from Sigma) were added at the following concentrations: for *E. coli*, ampicillin, 75 μg/ml; kanamycin, 25 μg/ml and spectinomycin, 50 μg/ml; for *S. suis*, spectinomycin, 100 μg/ml.

### RNA isolation and RT-PCR analysis

Total RNA samples were prepared from *S. suis* cultures using an SV total RNA isolation system (Promega), according to the manufacturer’s protocol. RNA concentrations and integrity were determined by UV spectrophotometry and agarose gel electrophoresis, respectively. RT-PCR was carried out using a QuantiTect Reverse Transcription Kit (Qiagen), according to the manufacturer’s instructions. For the co-transcription assay, the gene specific primers A1, A2, T1 and T2 were used for RT-PCR analysis (see Supplementary Table S2 online). To identify the mutant and complementation strains, primer pair ATin1/ATin2 was used.

### Plasmid Construction

Plasmids were constructed as follows using the primers listed in Supplementary Table S2.

1. pETBAD. Primer pair BAD1/BAD2 amplified the DNA fragment containing the *araC* gene and the promoter *P*_*BAD*_ from the pBADhisA plasmid. The DNA fragment was digested with the *Xho* I and *Hin*d III enzymes, and cloned into pET-30a, to generate the selective expression plasmid pETBAD.
2. pBADhisA-*yefM* and pBADhisA-*yoeB*. The *yefM* gene was amplified from the *S. suis* 2 genome using primer pair yefM1/yefM2. The PCR product was digested with the *Xho* I and *Hin*d III enzymes, and then cloned into pBADhisA, to generate plasmid pBADhisA-*yefM*. Plasmid pBADhisA-*yoeB* was constructed in a similar manner.
3. pBADhisA-*yefM-yoeB* and pET30a–*yefM-yoeB*. The *yefM* and *yoeB* genes were amplified from the *S. suis* 2 genome using primer pairs yefM1/R1 and R2/yoeB2, respectively. The two DNA fragments were fused into one fragment using overlap extension PCR. This DNA fragment was digested with the *Xho* I and *Hin*d III enzymes, and then ligated into pBADhisA, to generate pBADhisA-*yefM-yoeB*. Plasmid pET30a–*yefM-yoeB* was constructed in a similar manner, except that the *yefM* gene was amplified using primer pair yefM3/R1 and the fused DNA fragment was digested with the *Bam*H I and *Hind* III enzymes.
4. pETBAD-*yefM*_*Ssu*_*-yoeB*, pETBAD-*yefM*_*Spn*_*-yoeB* and pETBAD-*yefM*_*Eco*_*-yoeB*. The *yefM*_*Ssu*_ gene was amplified from the *S. suis* 2 genome using primer pair SsA1/SsA2. The DNA fragment was digested with the *Kpn* I and *Eco*R I enzymes, and then cloned into plasmid pETBAD to yield pETBAD-*yefM*. The *yoeB* gene was amplified from the *S. suis* 2 genome using primer pair SsT1/SsT2. After digestion with the *Hin*d III and *Sac* I enzymes, the fragment was cloned into pETBAD-*yefM*, to generate pETBAD-*yefM-yoeB*. This construct placed the *yefM*_*Ssu*_ and *yoeB* genes under the control of the IPTG-inducible promoter *P*_*lac*_ and the arabinose-inducible promoter *P*_*BAD*_, respectively. Thus, IPTG could induce the expression of YefM and arabinose could induce YoeB. The other two plasmids were constructed using the same procedure, except that the *yefM*_*Spn*_ and *yefM*_*Eco*_ genes were amplified from the *S. pneumoniae* R6 and *E. coli* K12 genomes, respectively.
5. pSET2-*yefM-yoeB*. A DNA fragment containing the *yefM-yoeB* locus and its predicted promoter was amplified from the *S. suis* 2 genome using primer pair CAT1/CAT2. After digestion with the *Pst* I and *Eco*R I enzymes, the fragment was cloned into pSET2, to generate the plasmid pSET2-*yefM-yoeB*.
6. pSET4s-Δ*yefM-yoeB*. Two flanking fragments (LA and RA) of the *yefM-yoeB* locus were amplified from the *S. suis* 2 genome using primer pairs LA1/LA2 and RA1/RA2, respectively. After digestion with the appropriate restriction enzymes, the two fragments were simultaneously cloned into pSET4s to generate the knockout plasmid pSET4s-Δ*yefM-yoeB*.

### *E. coli* growth analysis

*E. coli* Top10 cells transformed with pBADhisA-*yefM*, pBADhisA-*yoeB*, pBADhisA-*yefM-yoeB* and pBADhisA were cultured overnight in LB broth supplemented with 75 μg/mL ampicillin and 0.2% D-glucose. The next day, the four cultures were diluted 1:1000 in LB-ampicillin. Each culture was then divided into two equal volumes. The first half served as the control, to which 0.2% D-glucose was added, while 0.2% L-arabinose was added to the second half to induce expression of the target gene. Culture growth was evaluated by measuring the OD_600_ every hour.

*E. coli* BL21(DE3) cells harbouring the respective selective expression plasmids were incubated in LB broth supplemented with 25 μg/ml kanamycin to an OD_600_ of about 0.3. Each culture was divided into four equal parts, to three of which was individually added 0.2% L-arabinose, 1 mM IPTG or both, respectively. The fourth part had nothing added to it and served as a control. These cultures were further incubated and samples were taken every hour to determine the OD_600_.

*E. coli* strains were transformed with plasmids containing the *S. suis yefM-yoeB* system or the corresponding empty plasmids. Cells were cultured overnight in LB broth supplemented with antibiotics and diluted 1:1000 in fresh medium. Culture growth was monitored by measuring the OD_600_. For those cells showing growth arrest in LB broth, such as Top10 carrying pSET2-*yefM-yoeB*, isolated colonies were used as inocula. In parallel, colonies of the same size for strains carrying the corresponding empty plasmids were used.

### Deletion of the *yefM-yoeB* locus and functional complementation

Gene knockout mutant of the *yefM-yoeB* locus was constructed using plasmid pSET4s as described previously^47^,^48^. The knockout plasmid pSET4s-Δ*yefM-yoeB* was introduced into the competent cells of *S. suis* SC19 by electroporation. After two steps of allelic exchange at 28 °C, spectinomycin-sensitive clones were selected to identify the mutants by PCR using primers listed in Supplementary Table S2. The mutants were further confirmed by RT-PCR analysis and direct DNA sequencing.

For complementation assays, the recombinant plasmid pSET2-*yefM-yoeB* was introduced into the Δ*yefM-yoeB* mutant by electroporation. The complementation strain CΔ*yefM-yoeB* was selected with spectinomycin and confirmed using PCR, RT-PCR and DNA sequencing.

### Mouse infections

The Laboratory Animal Monitoring Committee of Huazhong Agricultural University approved all the animal experiments, which were performed in strict accordance with the recommendations in the Guide for the Care and Use of Laboratory Animals of Hubei Province, China. Forty female CD1 mice (5-weeks-old) were randomly divided into four groups with 10 mice per group. Mice in Groups 1, 2 and 3 were inoculated intraperitoneally with 6 × 10^8^ CFU in 200 μL PBS of the WT, Δ*yefM-yoeB* and CΔ*yefM-yoeB* strains, respectively. Group 4 was injected with 200 μL PBS, and served as the control group. Mice were monitored daily over 14 days for clinical signs and survival rates.

For the competitive-infection assay, four mice were inoculated intraperitoneally with a mixture of the WT and mutant strains at a ratio of 1:1 (1 × 10^8^ CFU). The actual ratio in the inoculum was determined by plating the suspension of each strain before mixing. Mice were sacrificed 24 h after inoculation and brain, heart, liver, spleen, lung and kidney samples were collected, homogenized and diluted for plating. Blood samples were directly diluted for plating. The Δ*yefM-yoeB*:WT ratios in these samples were determined by analysing 70–90 colonies using colony PCR with primer pair ATout1/ATout2, which yielded PCR products of 597 bp and 1117 bp for the Δ*yefM-yoeB* and WT strains, respectively. The competitive index (CI) was calculated as the Δ*yefM-yoeB*:WT ratio in each sample divided by the ratio in the inoculum.

### Bioinformatics and statistical analysis

Multiple sequence alignments were processed using ClustalW2 (http://www.ebi.ac.uk/Tools/msa/clustalw2/), and the modeled structures of *S. suis* YefM and YoeB were generated using CPHmodels 3.2 Server (http://www.cbs.dtu.dk/services/CPHmodels), which searches for templates from known structures. The promoter of the *yefM-yoeB* locus was predicted by BPROM (http://linux1.softberry.com/berry.phtml).

Statistical analyses were carried out using GraphPad Prism 5 (San Diego, USA). Log-rank test was used to analyse the mice survival curves. Two-tailed paired *t* test was used to analyse the data in the competitive-infection assay. A *P* value of <0.05 was considered statistically significant.

## Additional Information

**How to cite this article**: Zheng, C. *et al*. Identification and characterization of the chromosomal *yefM-yoeB* toxin-antitoxin system of *Streptococcus suis*. *Sci. Rep*. **5**, 13125; doi: 10.1038/srep13125 (2015).

## Supplementary Material

Supplementary Information
