# Supplementary material for: Identification and characterization of the chromosomal yefM-yoeB toxin-antitoxin system of Streptococcus suis
Source: Sci Rep. 2015 Aug 14;5:13125. doi: 10.1038/srep13125 (PMC4536659; doi:10.1038/srep13125)
Supplement: Supplementary Information [file srep13125-s1.doc]

**Supplementary Information**

**Identification and characterization of the** **chromosomal** ***yefM-yoeB* toxin-antitoxin system of *Streptococcus suis***

Chengkun Zheng ^1, 2, #^, Jiali Xu ^1, 2, #^, Sujing Ren ^1, 2^, Jinquan Li ^3^, Miaomiao Xia ^1^, Huanchun Chen ^1, 2^, Weicheng Bei ^1, 2^, *

^1^ State Key Laboratory of Agricultural Microbiology, College of Veterinary Medicine, Huazhong Agricultural University, Wuhan, Hubei, 430070, China

^2^ Key Laboratory of Development of Veterinary Diagnostic Products, Ministry of Agriculture, Huazhong Agricultural University, Wuhan, Hubei, 430070, China

^3^ College of Food Science and Technology, Huazhong Agricultural University, Wuhan, Hubei, 430070, China

# These authors contributed equally to this work.

* Correspondence and requests for materials should be addressed to W.B. (beiwc@mail.hzau.edu.cn)

**Supplementary Figures**

**Supplementary Figure S1. Phylogenetic tree of the *yefM-yoeB* locus in *S. suis* strains.**


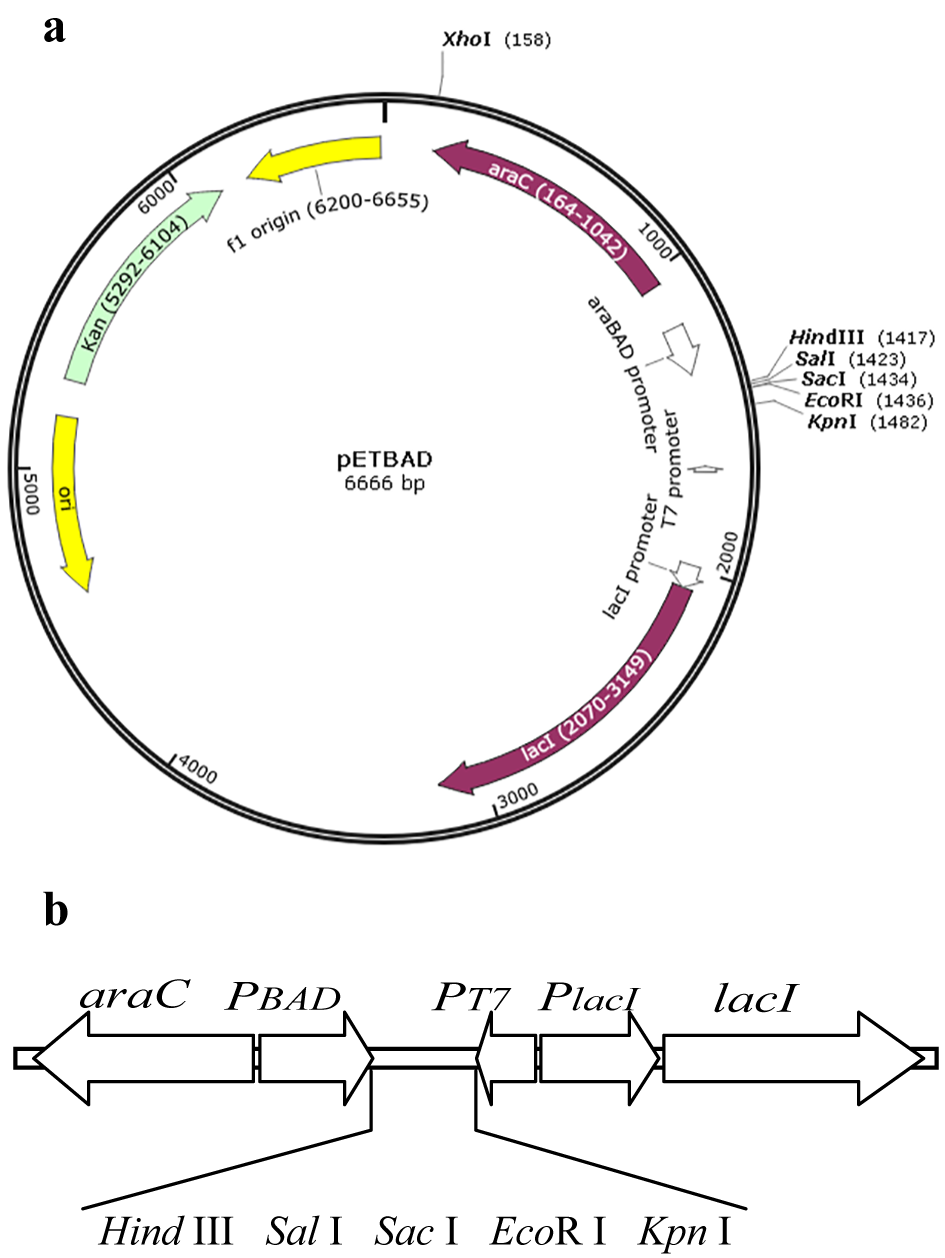


**Supplementary Figure S2. Schematic representation of the selective expression vector pETBAD.** (**a**) A DNA fragment containing the *araC* gene and the promoter *P_BAD_* was cloned into pET-30a to generate pETBAD. (**b**) The plasmid pETBAD has five unique restriction sites for cloning. Arrows indicate the direction of gene transcription and do not represent the exact length.


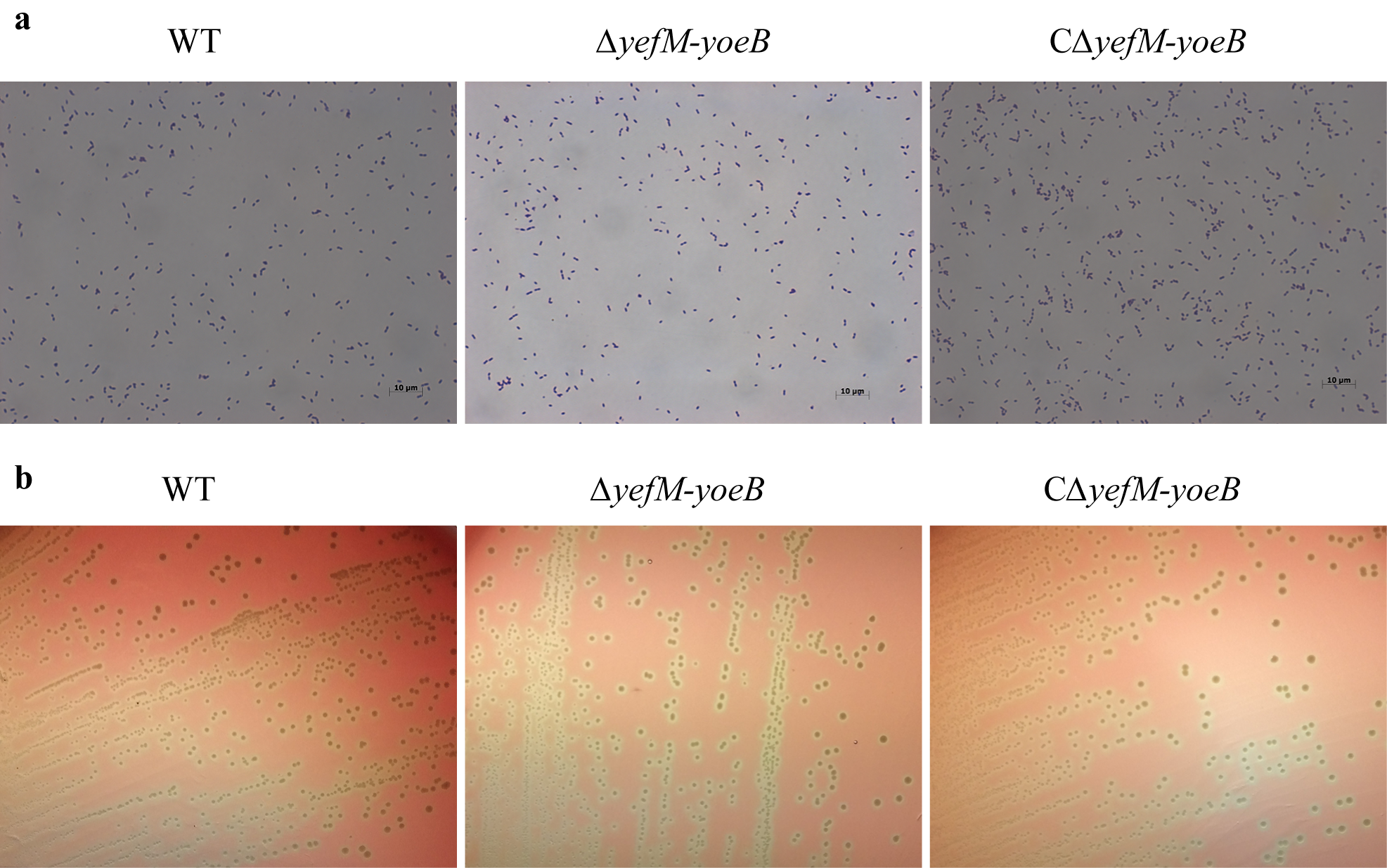


**Supplementary Figure S3. Cell morphology and hemolytic-activity analysis of the WT, *∆yefM-yoeB* and C*∆yefM-yoeB* strains.** (**a**) Morphology of *S. suis* strains under the light microscope using Gram staining. Bars, 10 μm. (**b**) Hemolytic activities of *S. suis* strains streaked on TSA plates containing 6% sheep blood and incubated for 24 h at 37°C.

**Supplementary Tables**

**Supplementary Table S1.** Bacterial strains and plasmids used in this study.

| Strain or plasmid | Relevant characteristics^a^ | Source or reference |
| --- | --- | --- |
| Strains |  |  |
| *S. suis* |  |  |
| SC19 | Virulent strain isolated from the brain of a dead pig; Serotype 2 | Laboratory collection |
| *∆yefM-yoeB* | The *yefM-yoeB* deletion mutant of SC19 | This study |
| C*∆yefM-yoeB* | Complemented strain *of ∆yefM-yoeB*; Spc^R^ | This study |
| *E. coli* |  |  |
| Trans5α | Cloning host for recombinant vector | TransGen |
| TOP10 | The expression host for pBADhisA and its derivative | TransGen |
| BL21 (DE3) | The expression host for pET-30a, pETBAD and their derivative | TransGen |
| Plasmids |  |  |
| pBADhisA | Expression vector; Amp^R^ | Invitrogen |
| pBADhisA-*yefM* | pBADhisA containing the *yefM* gene | This study |
| pBADhisA-*yoeB* | pBADhisA containing the *yoeB* gene | This study |
| pBADhisA-*yefM-yoeB* | pBADhisA containing the *yefM-yoeB* locus | This study |
| pET-30a | Expression vector; Kan^R^ | Novagen |
| pETBAD | Selective expression vector with the pET-30a background; Kan^R^ | This study |
| pETBAD-*yefM_Ssu_-yoeB* | pETBAD containing the *yefM_Ssu_* and *yoeB* genes | This study |
| pETBAD-*yefM_Spn_-yoeB* | pETBAD containing the *yefM_Spn_* and *yoeB* genes | This study |
| pETBAD-*yefM_Eco_-yoeB* | pETBAD containing the *yefM_Eco_* and *yoeB* genes | This study |
| pET30a-*yefM-yoeB* | pET30a containing the *yefM-yoeB* locus | This study |
| pSET2 | *E. coli*-*S. suis* shuttle vector; Spc^R^ | 41 |
| pSET2-*yefM-yoeB* | pSET2 containing the *yefM-yoeB* locus and its promoter | This study |
| pSET4s | *E. coli*-*S. suis* shuttle vector; Spc^R^ | 47 |
| pSET4s-*∆yefM-yoeB* | Knockout vector with the pSET4s background, designed for knockout of the *yefM-yoeB* locus | This study |

**Supplementary Table S2.** Primers used in this study.

| Primer | Sequence (5'-3')^a^ | Product size (bp) | Target gene |
| --- | --- | --- | --- |
| A1 | ATGGAAGCTATTGTATATTCCCATT | 261 | *yefM* |
| A2 | TTAGTCCGCCTCTATCAGGTTT |  |  |
| T1 | ATGGGAATCCATTTTACAGACG | 258 | *yoeB* |
| T2 | TCACCTATAATGATCCTTTAGCGA |  |  |
| BAD1 | CCGCCTCGAGTTATGACAACTTGACGGCTACA | 1253 | *araC* and *P_BAD_* |
| BAD2 | CCCCAAGCTTATGATGATGATGATGATGAGAAC |  |  |
| yefM1 | CCGCCTCGAGATGGAAGCTATTGTATATTCCCATT | 261 | *yefM* |
| yefM2 | CCCCAAGCTTTTAGTCCGCCTCTATCAGGTTT |  |  |
| yoeB1 | CGGGGTACCATATGGGAATCCATTTTACAGACG | 258 | *yoeB* |
| yoeB2 | CCCCAAGCTTTCACCTATAATGATCCTTTAGCGA |  |  |
| R1 | CGTCTGTAAAATGGATTCCCATTTAGTCCGCCTCTATCAGGTTT |  |  |
| R2 | ATGGGAATCCATTTTACAGACG |  |  |
| SsA1 | CGGCGGTACCATGGAAGCTATTGTATATTCCCAT | 261 | *yefM* |
| SsA2 | CGCCGAATTCTTAGTCCGCCTCTATCAGGTT |  |  |
| SsT1 | CCCCAAGCTTATGGGAATCCATTTTACAGAC | 258 | *yoeB* |
| SsT2 | CCGCGAGCTCGATCACCTATAATGATCCTTTAGCG |  |  |
| SpA1 | CGGCGGTACCGTGTATAATAGTGGAAAAGAGCTAAAAC | 294 | *yefM_Spn_* |
| SpA2 | CGCCGAATTCTCACTCCTCAATCACATGGAC |  |  |
| EcA1 | CGGCGGTACCATGCGTACAATTAGCTACAGC | 252 | *yefM_Eco_* |
| EcA2 | CGCCGAATTCTCACTCAATGATGTCCTTTTC |  |  |
| yefM3 | CGCCGGATCCATGGAAGCTATTGTATATTCCCATT |  |  |
| CAT1 | AAAACTGCAGTCTCGAACTCTGTGGCATTC | 857 | *yefM-yoeB* and its promoter |
| CAT2 | CGCCGAATTCTCACCTATAATGATCCTTTAGCG |  |  |
| LA1 | CGCCAAGCTTATGTAGTAGGGCAGGTTGGC | 952 | Left arm of *yefM-yoeB* |
| LA2 | CCCCGTCGACGGGGGATTAACAAATGAAAAA |  |  |
| RA1 | CCCCGTCGACAAGCAATACCTCTCATCATTTCTAG | 857 | Right arm of *yefM-yoeB* |
| RA2 | CCGCGAGCTCAGTCTTATTCAGTGATACTTGCTCC |  |  |
| ATin1 | ATCAGCTTCCACCATATAAATCA | 434 | an internal fragment of *yefM-yoeB* |
| ATin2 | TATGAAAAAGGTCAATGACGAGT |  |  |
| ATout1 | ACTGCTTGTGTTAACTCCATCTG | 1117/597 | a fragment containing *yefM-yoeB* |
| ATout2 | TGTGGCATTCAAAACTGGAC |  |  |

^a^ The underlined sequences are restriction sites.
